# Navigating Compassion: A Comprehensive Review of Palliative Care in Respiratory Medicine

**DOI:** 10.7759/cureus.50613

**Published:** 2023-12-16

**Authors:** Ulhas Jadhav, Jay Bhanushali, Arman Sindhu, Bingu Shiv Kiran Reddy

**Affiliations:** 1 Respiratory Medicine, Jawaharlal Nehru Medical College, Datta Meghe Institute of Higher Education and Research, Wardha, IND

**Keywords:** end-of-life care, caregiver support, symptom management, chronic obstructive pulmonary disease (copd), respiratory medicine, palliative care

## Abstract

Palliative care has emerged as a crucial aspect of comprehensive healthcare, particularly in respiratory medicine. This review navigates the intricate landscape of palliative care in the context of respiratory diseases, including chronic obstructive pulmonary disease (COPD), idiopathic pulmonary fibrosis (IPF), and lung cancer. The exploration begins with a comprehensive examination of palliative care's definition, significance, and purpose in respiratory medicine. It progresses to understanding common respiratory diseases, their impact on patients' quality of life, and the nuances of disease progression and prognosis. Delving into the principles of palliative care, the review highlights the importance of a patient- and family-centered approach, emphasizing the multidisciplinary collaboration required for holistic care. Symptom management takes center stage, with a detailed exploration of dyspnea, cough, and pain, covering pharmacological and non-pharmacological interventions. The psychosocial and spiritual dimensions are then unveiled, recognizing the psychological impact of respiratory diseases and the significance of addressing spiritual needs with cultural sensitivity. Communication in palliative care is explored through breaking lousy news, advance care planning, and shared decision-making. The section acknowledges the complex considerations surrounding end-of-life care, including recognizing the end-of-life phase, establishing care goals, and withdrawing life-sustaining therapies. Recognizing the indispensable role of caregivers, the review underscores the importance of caregiver support. It delineates strategies for providing emotional and practical support alongside a crucial focus on self-care for caregivers who shoulder the responsibilities of providing palliative care. As the exploration concludes, the challenges in implementing palliative care in respiratory medicine are outlined, from late referrals to communication barriers. However, the review also envisions a future marked by innovation, with emerging approaches, such as telehealth and personalized medicine, offering promising avenues for improvement. Research gaps and areas for improvement are identified, emphasizing the need for a collaborative effort to enhance the quality of palliative care for individuals facing respiratory diseases. The review culminates in a call to action, urging early palliative care integration, investment in education and training, research initiatives, advocacy for accessible services, and collaboration across disciplines. By heeding this call, healthcare providers, researchers, and policymakers can collectively contribute to the evolution and enhancement of palliative care in the challenging landscape of respiratory medicine.

## Introduction and background

Palliative care, a discipline rooted in compassionate and holistic patient-centered care, has become increasingly recognized as essential in managing complex and life-limiting illnesses. Within medical specialties, respiratory medicine stands out as a field where the integration of palliative care is paramount [1]. Palliative care is a specialized approach to healthcare that focuses on improving the quality of life for patients and their families facing life-threatening illnesses. It encompasses a holistic model of care that addresses not only the physical symptoms but also attends to individuals' psychosocial, spiritual, and emotional needs. In respiratory medicine, palliative care goes beyond traditional disease-focused treatments to enhance the overall well-being of patients grappling with chronic and often progressive pulmonary conditions [2].

Palliative care seeks to alleviate suffering by managing symptoms, fostering effective communication, and supporting patients in making informed decisions about their care. It is not synonymous with end-of-life care; instead, it is a dynamic and integrated approach that can be implemented at any stage of a respiratory illness, from the time of diagnosis throughout the trajectory of the disease [3]. The significance of integrating palliative care into the fabric of respiratory medicine lies in its ability to enhance the quality of life for patients facing the challenges of diseases, such as chronic obstructive pulmonary disease (COPD), idiopathic pulmonary fibrosis (IPF), and lung cancer. Respiratory diseases often bring with them a burden of debilitating symptoms, breathlessness, and a decline in overall functioning, making the role of palliative care pivotal in addressing these issues [4]. Furthermore, palliative care in respiratory medicine is essential in navigating the complex decision-making processes associated with advanced directives, goals of care, and end-of-life discussions. The incorporation of palliative care principles ensures that patients receive care that aligns with their values and preferences, fostering a sense of control and dignity amid the challenges posed by respiratory illnesses [5].

This comprehensive review aims to provide an in-depth exploration of palliative care within the context of respiratory medicine. By examining the principles, challenges, and advancements in this field, we aim to contribute to understanding how palliative care can be optimally integrated into the care continuum for individuals facing respiratory diseases. This review aspires to offer a valuable resource for healthcare professionals, researchers, and policymakers invested in improving the holistic care of patients with respiratory conditions through a critical analysis of existing literature, practical insights, and emerging trends. As we navigate the intricate landscape of compassion in respiratory medicine, our goal is to shed light on the pivotal role of palliative care in enhancing the lives of individuals confronting these challenging illnesses.

## Review

Understanding respiratory diseases

Overview of Common Respiratory Diseases

COPD: COPD is a prevalent respiratory condition characterized by persistent airflow limitation. Often caused by exposure to noxious particles or gases, such as those found in cigarette smoke, COPD encompasses conditions, including chronic bronchitis and emphysema. The chronic and progressive nature of COPD significantly impacts patients' respiratory function, leading to symptoms, such as breathlessness, chronic cough, and increased susceptibility to respiratory infections [6].

IPF: IPF is a chronic and irreversible interstitial lung disease characterized by the progressive scarring of the lung tissue. The exact cause of IPF remains unknown, and its diagnosis often comes after excluding other potential causes of pulmonary fibrosis. As the disease advances, patients experience worsening dyspnea, a decline in lung function, and impaired oxygen exchange. IPF poses significant challenges due to its unpredictable progression and limited treatment options [7].

Lung cancer: Lung cancer, a leading cause of cancer-related mortality worldwide, encompasses a range of malignancies originating in the lung tissue. It is often associated with a history of tobacco use, although non-smokers can also be affected. Lung cancer's impact extends beyond the lungs, affecting systemic health and quality of life. Symptoms may include persistent cough, hemoptysis, and respiratory distress. The prognosis varies based on the type and stage of lung cancer at the time of diagnosis [8].

Impact on Patients' Quality of Life

The burden of respiratory diseases on patients' quality of life is profound and multifaceted. Chronic symptoms, such as breathlessness, coughing, and fatigue, can significantly limit physical activities and daily functioning. The psychological impact is equally significant, with patients often experiencing anxiety and depression due to the chronic nature and uncertainty associated with respiratory illnesses. The progressive nature of diseases, such as COPD and IPF, adds a layer of complexity, impacting not only the patients but also their families and caregivers [9].

Disease Progression and Prognosis

Understanding the trajectory of respiratory diseases is crucial for healthcare providers and patients alike. Disease progression varies among individuals and is influenced by factors, such as the underlying cause, comorbidities, and response to treatment. Prognosis assessment involves considering the stage of the disease, functional impairment, and the presence of complications. Providing patients and their families with accurate and realistic information about the expected course of the disease is essential for fostering informed decision-making and facilitating timely discussions about palliative care options. In the subsequent sections, we will delve into how palliative care can play a pivotal role in addressing the unique challenges posed by the progression of respiratory diseases [10].

Palliative care principles

Integration of Palliative Care in Respiratory Medicine

Integrating palliative care into respiratory medicine is crucial for optimizing patient outcomes and experiences. Palliative care principles should be seamlessly woven into the fabric of care from the point of diagnosis throughout the trajectory of the respiratory disease. Early integration ensures that patients and their families receive the necessary support to cope with the physical and emotional challenges associated with conditions, such as COPD, IPF, and lung cancer. This integration is not about replacing curative treatments but enhancing the overall care experience, promoting shared decision-making, and aligning interventions with patients' values and preferences [11].

Multidisciplinary Approach

Palliative care in respiratory medicine necessitates a multidisciplinary approach that brings together healthcare professionals with diverse expertise to address the complex needs of patients. This may include pulmonologists, nurses, respiratory therapists, social workers, psychologists, and spiritual care providers. The collaboration of these specialists ensures a comprehensive assessment of the patient's physical and psychosocial needs and facilitates a coordinated effort to implement tailored interventions. The multidisciplinary approach is essential for delivering holistic care that acknowledges the diverse challenges of individuals with respiratory diseases [12].

Patient and Family-Centered Care

At the heart of palliative care in respiratory medicine is the principle of patient and family-centered care. Recognizing that illness affects not only the individual but also their loved ones, palliative care actively involves patients and their families in decision-making processes. Open and honest communication, shared decision-making, and sensitivity to cultural and individual values are integral components of patient and family-centered care. This approach empowers patients to actively participate in their care actively, fostering a sense of control and dignity amid the complexities of respiratory diseases [13].

Symptom management in respiratory patients

Dyspnea

Dyspnea, characterized by difficulty breathing, is a hallmark symptom in various respiratory diseases, emphasizing the significance of understanding its underlying causes for targeted management. The causes of dyspnea can span bronchoconstriction, lung parenchymal changes, or heightened respiratory effort. Assessment of dyspnea involves a thorough examination, encompassing the patient's medical history, a physical examination, and, when necessary, imaging studies and pulmonary function tests. Pharmacological interventions are vital in managing dyspnea, with options, including bronchodilators (beta-agonists and anticholinergics), corticosteroids, and oxygen therapy [14]. Bronchodilators work to alleviate bronchoconstriction and enhance airflow, while corticosteroids, particularly in conditions, such as COPD, address inflammation. Oxygen therapy, when indicated, aims to improve oxygen saturation and alleviate dyspnea. Non-pharmacological interventions are equally pivotal, incorporating pulmonary rehabilitation, breathing exercises, and relaxation techniques to enhance respiratory muscle function and overall capacity. Supportive measures, such as positioning, fan therapy, and activity pacing, further contribute to relieving dyspnea, presenting a comprehensive approach to its management [14].

Cough

Cough, a prevalent symptom in various respiratory diseases, can be attributed to diverse factors, such as airway inflammation, irritants, or tumors. Assessing the root cause of a cough is pivotal and typically involves a comprehensive examination encompassing a detailed clinical history, a thorough physical examination, and, when deemed necessary, the utilization of imaging studies. Identifying the underlying factors contributing to cough is crucial for effective management in respiratory medicine [15]. Regarding treatment options, the approach aims to address the specific cause of the cough. In respiratory medicine, interventions may include bronchodilators to alleviate airway constriction, mucolytic agents to facilitate the thinning of mucus for more straightforward clearance, or antitussive medications when the cough becomes particularly distressing. Tailoring treatment to the individual's specific condition underscores the importance of a personalized and targeted approach in managing cough as a symptom of respiratory diseases [15].

Pain Management

Pain in respiratory patients can emanate from diverse sources, encompassing chest wall pain, pleuritic pain, or discomfort associated with various procedures and interventions. Identifying the specific source of pain is paramount for tailoring effective and targeted pain management strategies. Chest wall pain may be related to musculoskeletal issues or inflammation, while pleuritic pain often results from inflammation of the pleura. Pain associated with medical procedures and interventions, such as surgery or diagnostic tests, further adds to the complexity of pain management in this population [16]. Analgesic strategies employed in respiratory medicine embrace a multifaceted approach, combining pharmacological and non-pharmacological interventions. Pharmacological options include the use of nonsteroidal anti-inflammatory drugs (NSAIDs), opioids, and adjuvant medications to address pain stemming from different origins. Non-pharmacological interventions are complementary, involving physical therapy to enhance musculoskeletal function, breathing exercises to improve respiratory function, and relaxation techniques to alleviate overall discomfort. This comprehensive approach to pain management recognizes the multifaceted nature of pain in respiratory patients, striving to enhance their overall well-being through personalized and effective interventions [16].

Psychosocial and spiritual dimensions

Psychological Impact of Respiratory Diseases

Anxiety and depression: Respiratory diseases often evoke anxiety and depression due to the challenges associated with chronic symptoms, reduced functional capacity, and uncertainty about the future. Anxiety may arise from the fear of breathlessness or exacerbations, while depression can result from the impact of the disease on daily life and social interactions. Screening tools and open communication with patients can help identify these psychological challenges [17].

Coping mechanisms: Coping with respiratory disease's psychological impact involves individual and support-based strategies. Patients benefit from education about their condition, support groups, and counseling services. Cognitive-behavioral therapy (CBT) and mindfulness techniques can be effective in helping patients develop coping mechanisms and resilience in the face of the emotional challenges posed by respiratory illnesses [18].

Addressing Spiritual Needs

Importance of spiritual care: Spiritual care recognizes the importance of addressing the existential and spiritual dimensions of an individual's experience with illness. In respiratory medicine, acknowledging and respecting patients' spiritual beliefs and values is integral to providing holistic care. Spiritual care contributes to a sense of meaning, purpose, and connectedness, which can positively impact overall well-being, even in the face of chronic and progressive diseases [19].

Cultural sensitivity: Cultural sensitivity in addressing spiritual needs is paramount. Different cultures have diverse beliefs and practices about illness, death, and spirituality. Healthcare providers should engage in open and respectful conversations with patients to understand their cultural context. This may involve collaboration with spiritual or religious leaders when appropriate, ensuring that care aligns with the patient's cultural and spiritual preferences [20].

Communication in palliative care

Breaking Bad News

Breaking bad news is a delicate and crucial aspect of palliative care, especially in respiratory medicine, where conditions can be chronic and progressive. Communication should be honest, empathetic, and tailored to the patient's needs. This involves providing information clearly and understandably, gauging the patient's readiness to receive information, and addressing emotional responses. Supporting patients and their families through bad news discussions is vital for building trust and facilitating informed decision-making [21].

Advance Care Planning

Advanced care planning involves discussing and documenting an individual's preferences and values regarding their future healthcare, especially in a potential decline in health or incapacity. This process empowers patients to make decisions about their care that align with their values. In respiratory medicine, advance care planning may include discussions about intubation, mechanical ventilation, and preferences for end-of-life care. These conversations should be ongoing, revisited regularly, and documented in the patient's medical record [22].

Shared Decision-Making

Shared decision-making is a collaborative approach where healthcare providers and patients work together to make healthcare decisions. In respiratory medicine, shared decision-making is critical due to the complex nature of treatment options, potential side effects, and the impact on the patient's quality of life. This approach involves presenting information about treatment options, discussing potential benefits and risks, and considering the patient's values and preferences. Shared decision-making fosters a partnership between healthcare providers and patients, ensuring that care aligns with the individual's goals and priorities [23].

Addressing Cultural and Ethical Considerations

Cultural and ethical considerations play a significant role in communication within the palliative care context. Recognizing and respecting diverse cultural beliefs and values related to illness, death, and decision-making is essential. Healthcare providers should approach conversations with cultural humility, acknowledging their biases and actively seeking to understand the cultural context of each patient. Ethical considerations involve ensuring autonomy, beneficence, and non-maleficence in decision-making processes. This includes navigating issues related to withdrawing life-sustaining treatments and ensuring that decisions align with the patient's values and ethical principles [24].

End-of-life care in respiratory medicine

Recognizing the End-of-Life Phase

Clinical indicators: Recognizing the end-of-life phase in respiratory medicine involves assessing clinical indicators that suggest a decline in health. This may include a progressive decrease in functional capacity, increased symptom burden, recurrent infections, and a trajectory of irreversible decline despite interventions. Regular assessments, including discussions with the patient and their family, can aid in identifying when the end-of-life phase is approaching [25].

Prognostication challenges: Prognostication in respiratory diseases can be challenging due to the unpredictable nature of conditions, such as COPD, IPF, and lung cancer. Healthcare providers should exercise caution in predicting precise timelines but can provide general information about the expected trajectory based on the patient's disease characteristics [26].

Goals of Care at the End of Life

Establishing patient-centered goals: End-of-life care in respiratory medicine demands a patient-centered approach, recognizing the unique values and priorities of the individual facing a life-limiting illness. This involves collaborative discussions among the patient, their family, and the healthcare team to identify goals aligning with their wishes. By fostering open communication, healthcare providers can gain insights into patients' aspirations, concerns, and what matters most to them. Realistic and achievable goals are formulated, emphasizing aspects, such as maximizing comfort, preserving dignity, and facilitating meaningful interactions with loved ones. This patient-centered goal-setting process ensures that the care provided is tailored to the individual, enhancing their overall quality of life during the challenging end-of-life phase [27].

Advance directives and documentation: Advance care planning is a crucial component of end-of-life care in respiratory medicine, involving the documentation of the patient's preferences and goals for their healthcare. Advance directives and living will serve as essential documents that articulate the patient's choices regarding medical interventions, resuscitation preferences, and broader care goals. These documents provide a roadmap for healthcare decisions when patients cannot communicate their wishes. Ensuring that these documents are in place and up-to-date is imperative. Clear communication of the documented preferences to the healthcare team guarantees that medical decisions align with the patient's established values and priorities. This comprehensive approach to advance directives and documentation empowers individuals to have a say in their care, even in times of incapacity and promotes a more informed and patient-centered decision-making process [22].

Withdrawal of Life-Sustaining Therapies

Transparent communication: Discussions surrounding the withdrawal of life-sustaining therapies demand a communication approach characterized by transparency and empathy. Healthcare providers are tasked with openly addressing the complexities of this decision, ensuring that the patient and their family comprehend both the potential benefits and burdens associated with continuing or discontinuing interventions. Transparent communication facilitates informed decision-making, allowing the patient and their family to understand the implications of the choices ahead fully. This approach, grounded in openness and empathy, fosters trust between healthcare providers and patients or their surrogate decision-makers during a sensitive and challenging time [28].

Multidisciplinary collaboration: The decision to withdraw life-sustaining therapies is multifaceted and requires input from various healthcare professionals. A multidisciplinary team encompassing respiratory therapists, nurses, social workers, and palliative care specialists collaborates to ensure a comprehensive and well-informed decision-making process. Each team member brings a unique perspective and expertise, contributing to a holistic understanding of the patient's medical, emotional, and social context. This collaborative effort ensures that the decision aligns with the patient's wishes, is compassionate, and respects the values and goals established by the patient and their family [29].

Psychosocial support: Initiating the withdrawal of life-sustaining therapies introduces a significant emotional burden for both patients and their families. Recognizing the emotional challenges inherent in this transition, the provision of psychosocial support becomes paramount. This support encompasses a range of services, including counseling, spiritual care, and bereavement support. Counseling services offer a space for individuals to express their feelings, fears, and uncertainties, providing emotional support during this difficult time. Spiritual care attends to the existential and spiritual dimensions, offering solace and meaning. Bereavement support helps individuals cope with grief and loss, recognizing that the decision to withdraw from life-sustaining therapies marks a profound and impactful moment in the patient's and family's journey. The ready availability of these psychosocial support services ensures that individuals facing this transition receive the comprehensive and compassionate care needed to navigate the emotional complexities of such a decision [30].

Caregiver support

Importance of Caregivers in Palliative Care

Integral to the care team: Caregivers are not mere observers but integral palliative care team members, contributing essential support to patients navigating respiratory diseases. Their role extends beyond physical assistance, encompassing emotional, psychological, and often spiritual dimensions of care. Recognizing the caregiver as a valued care team member is foundational for delivering truly holistic, patient-centered care. Caregivers bring unique insights into the daily experiences and needs of the patient, acting as a bridge between the healthcare team and the individual receiving care. Integrating their perspectives and expertise ensures a more comprehensive understanding of the patient's condition and facilitates tailored care that addresses the multifaceted challenges of respiratory diseases [31].

Enhancing patient well-being: Caregivers significantly influence the overall well-being of patients facing respiratory diseases. Their presence gives the patient a sense of security, comfort, and emotional stability. Beyond the physical tasks of caregiving, they often become advocates, ensuring that the care provided aligns with the individual's values, preferences, and wishes. Caregivers are attuned to the emotional and psychological needs of the patient, offering companionship and a supportive presence during challenging times. By actively engaging with caregivers and recognizing their contributions, healthcare providers can foster a collaborative and compassionate care environment that prioritizes the patient's well-being. This acknowledgment validates the essential role of caregivers and strengthens the patient-caregiver-healthcare team partnership, ultimately contributing to a more positive and patient-centered care experience [32].

Providing Emotional and Practical Support

Open communication: Establishing open and regular communication channels with caregivers is fundamental to providing comprehensive, patient-centered care. This involves ongoing discussions about the patient's condition and treatment plans and addressing caregivers' concerns or uncertainties. Transparent communication keeps caregivers informed about the patient's health status and fosters a sense of trust and collaboration between healthcare providers and caregivers. By maintaining open lines of communication, healthcare teams can ensure that caregivers are well-equipped to support the patient effectively, understand the rationale behind medical decisions, and actively participate in the care planning process [33].

Emotional support: Respiratory diseases pose emotional challenges for patients and their caregivers. Providing emotional support to caregivers is essential for addressing the psychological impact of caregiving. This support involves acknowledging the caregiver's feelings, offering empathetic listening, and validating the emotional toll that caring for someone with a respiratory illness can take. Healthcare providers play a crucial role in recognizing and addressing caregiver stress, anxiety, or grief. Access to counseling services or support groups can provide additional avenues for caregivers to express their emotions, share experiences, and receive guidance on coping strategies. By offering emotional support, healthcare providers contribute to the overall well-being of both the patient and caregiver [34].

Practical assistance: Caregivers often juggle various responsibilities, including complex caregiving tasks, such as medication management, assisting with activities of daily living, and coordinating medical appointments. Providing practical assistance is essential for helping caregivers navigate their roles effectively. This can include offering educational resources that provide information on disease management, organizing training sessions to enhance caregiving skills, and facilitating access to respite care services. Respite care allows caregivers to take breaks, recharge, and attend to their well-being. By addressing the practical aspects of caregiving, healthcare providers support caregivers in providing quality care to the patient while also attending to their own needs, ultimately contributing to a more sustainable and positive caregiving experience [35].

Self-Care for Caregivers

Recognizing burnout and stress: Caregiving is a demanding role that can affect caregivers' physical and emotional well-being, leading to burnout and stress. Recognizing the signs of burnout, such as fatigue, changes in mood, or a sense of overwhelm, is crucial for caregivers. Healthcare providers are vital in encouraging caregivers to be attentive to their mental and physical health. Regular check-ins and open communication channels with healthcare providers create opportunities to discuss caregiver stressors, identify early signs of burnout, and collaboratively develop strategies to manage and mitigate stress [36].

Promoting self-care practices: Educating caregivers about self-care is essential for maintaining their well-being. This involves promoting healthy lifestyle habits, ensuring caregivers prioritize adequate rest, and encouraging engagement in leisure activities. Caregivers often neglect their needs while caring for a loved one, and healthcare providers can offer guidance on incorporating self-care practices into their routines. Providing resources for respite care, where caregivers can take a temporary break from their caregiving responsibilities, and facilitating access to support groups create a supportive environment for caregivers to share experiences and learn strategies for managing their challenges [37].

Offering professional support: Acknowledging that caregiving may require professional assistance is crucial to caregiver support. Healthcare providers can be pivotal in connecting caregivers with additional resources and services. This may include home healthcare services to provide additional assistance with caregiving tasks, counseling services to address emotional and mental health needs and support groups where caregivers can connect with others facing similar challenges. Professional support acknowledges the complexity of the caregiving role and ensures that caregivers have access to the necessary resources to prevent burnout and maintain their well-being [38].

Challenges and future directions

Challenges in Implementing Palliative Care in Respiratory Medicine

Late referrals to palliative care: Late referrals to palliative care for respiratory patients pose a significant challenge, as individuals may need more comprehensive support that could enhance their quality of life throughout their disease. Overcoming this challenge involves addressing the reluctance among healthcare providers to initiate palliative care discussions early in the trajectory of respiratory diseases. Increasing awareness about the benefits of early palliative care, along with providing education to healthcare providers, can help shift the paradigm. By emphasizing the advantages of integrating palliative care from the point of diagnosis, healthcare professionals can work toward ensuring that individuals and their families receive timely and holistic support tailored to their evolving needs [39].

Communication barriers: Effective communication about palliative care, especially discussions related to end-of-life care, can be challenging for healthcare providers. Resistance may arise from patients, families, or even fellow professionals due to the sensitive nature of these conversations. To address these communication barriers, ongoing training programs are essential. Improving communication skills, including empathetic listening and discussing palliative care in a culturally sensitive manner, can enhance healthcare providers' ability to navigate these discussions. In addition, increasing public awareness about the benefits of early palliative care involvement can help normalize these conversations, making them more acceptable for all parties involved [40].

Limited access to palliative care services: Disparities in access to palliative care services, especially in rural or underserved areas, present a challenge in ensuring equitable care for all respiratory patients. Overcoming these disparities requires a multifaceted approach. Addressing logistical and resource challenges involves strategic planning to allocate resources efficiently and leveraging technology to provide virtual access to palliative care services. Expanding palliative care training for healthcare providers in various settings, including rural communities, can enhance the availability of these services. Advocating for policies prioritizing and enhancing the accessibility of palliative care ensures that individuals facing respiratory diseases, regardless of geographic location or socioeconomic status, have access to the support and care they need throughout their healthcare journey [41].

Innovations and Emerging Approaches

Telehealth and remote monitoring: Integrating telehealth and remote monitoring technologies represents a promising frontier in palliative care for respiratory patients. These innovations leverage digital tools to bridge geographical distances and enhance care delivery. Virtual consultations enable healthcare providers to connect with patients and their caregivers, offering timely assessments and interventions without the constraints of physical proximity. Remote monitoring capabilities allow for the continuous tracking of symptoms and vital signs, providing a real-time understanding of the patient's condition. This not only improves accessibility to care but also contributes to the continuity of support, enabling healthcare providers to respond promptly to changes in the patient's health status. By embracing telehealth and remote monitoring, palliative care for respiratory patients can become more patient-centered, adaptable, and responsive to the dynamic nature of their healthcare needs [42].

Personalized medicine in symptom management: Advancements in personalized medicine open new avenues for tailoring symptom management strategies in respiratory palliative care. By considering an individual's unique characteristics and genetic makeup, healthcare providers can design interventions that align with the patient's needs. Personalized approaches to symptom management not only optimize the effectiveness of interventions but also aim to minimize potential side effects. This level of customization enhances the overall patient experience by recognizing and addressing the variability in how individuals respond to treatments. As the field of personalized medicine evolves, it can revolutionize how respiratory disease symptoms are managed in palliative care, promoting a more targeted and patient-centric approach to symptom relief and overall care [43].

Research Gaps and Areas for Improvement

Understanding patient and caregiver perspectives: There is a critical need for research that delves into the perspectives of both patients and caregivers regarding palliative care in respiratory medicine. By gaining insights into their unique needs, preferences, and experiences, researchers can inform the development of more patient-centered interventions. Understanding the challenges and priorities from the viewpoint of those directly affected by respiratory diseases and palliative care allows for the creation tailored strategies that resonate with the individuals involved. This research validates the importance of patient and caregiver voices and ensures that palliative care interventions align with the values and preferences of those receiving and providing care [44].

Impact of palliative care on quality of life: Further research is essential to comprehensively assess the impact of palliative care on the quality of life for individuals grappling with respiratory diseases. This research involves evaluating the effectiveness of palliative care interventions, exploring the long-term outcomes for patients and their caregivers, and identifying the factors contributing to positive experiences. By rigorously examining the impact of palliative care, researchers can contribute valuable evidence that informs healthcare practices, policies, and interventions. Understanding the specific ways in which palliative care enhances the quality of life for respiratory patients is integral to fostering continuous improvement in the delivery of patient-centered care [45].

Education and training for healthcare providers: Research aimed at evaluating the effectiveness of educational and training programs for healthcare providers in palliative care is imperative. This research focus includes understanding the barriers that healthcare providers may encounter when implementing palliative care principles and identifying strategies to enhance their knowledge and skills. By assessing the impact of educational interventions, researchers can contribute to the ongoing refinement of training programs, ensuring that healthcare providers are well-equipped to integrate palliative care seamlessly into respiratory medicine. This research also plays a pivotal role in advocating for continuous education in palliative care, fostering a healthcare environment prioritizing delivering holistic, patient-centered care [46].

## Conclusions

Palliative care in respiratory medicine emerges as a crucial and evolving discipline that demands attention and proactive engagement. This comprehensive review has highlighted the significance of early integration, effective communication, and caregiver support in optimizing the quality of life for individuals facing conditions, such as COPD, IPF, and lung cancer. It underscores the need for a holistic approach beyond addressing physical symptoms to encompass caregivers' psychosocial and spiritual dimensions. As we navigate challenges, such as late referrals and communication barriers, there is a call to action for healthcare providers, policymakers, and researchers alike. Investment in education, advocacy for accessible services, and collaborative efforts across disciplines are essential for shaping the future of palliative care in respiratory medicine. By embracing innovation, addressing research gaps, and fostering a commitment to patient-centered care, we can collectively enhance the well-being of individuals and their families confronting the complexities of respiratory diseases.
